# Direct oral anticoagulants and warfarin in atrial fibrillation patients with cancer by anticoagulation quality

**DOI:** 10.1002/cncr.70501

**Published:** 2026-07-07

**Authors:** Danilo Menichelli, Vito Maria Daniele Cormaci, Gianluca Gazzaniga, Irma Bisceglia, Massimiliano Camilli, Daniela Poli, Emilia Antonucci, Roberto Pola, Paolo Santini, Pasquale Pignatelli, Daniele Pastori, the START2 Registry Investigators

**Affiliations:** ^1^ Department of General Surgery, Surgical Specialty and Anesthesiology Paride Stefanini, PhD School in Advances in Cardio‐Thoracic and Vascular Pathophysiology and Imaging Sapienza University of Rome Rome Italy; ^2^ Department of Medical and Cardiovascular Sciences Sapienza University of Rome Rome Italy; ^3^ Integrated Cardiology Services, Department of Cardio‐Thoracic‐Vascular Azienda Ospedaliera San Camillo Forlanini Rome Italy; ^4^ Department of Cardiovascular and Pulmonary Sciences Catholic University of the Sacred Heart Rome Italy; ^5^ Department of Cardiovascular Sciences CUORE Fondazione Policlinico Universitario A. Gemelli IRCCS Rome Italy; ^6^ Department of Critical Care Medicine Thrombosis Centre Azienda Ospedaliera Universitaria Careggi Florence Italy; ^7^ Arianna Anticoagulazione Foundation Bologna Italy; ^8^ Thrombosis Unit, Department of Aging, Orthopedic, and Rheumatologic Sciences Fondazione Policlinico Universitario A. Gemelli IRCCS Università Cattolica del Sacro Cuore Rome Italy; ^9^ IRCCS Neuromed Pozzilli (IS) Italy

**Keywords:** atrial fibrillation, cancer, cardiovascular events, direct oral anticoagulants, mortality

## Abstract

**Background:**

Atrial fibrillation (AF) patients with cancer are frequently treated with vitamin K antagonists (VKAs). Direct oral anticoagulants (DOACs) benefit according to VKA quality has not been investigated in this high‐risk population. The authors compared DOACs with VKAs on mortality, cardiovascular events (CVEs), and bleeding risk across time‐in‐therapeutic‐range (TiTR) strata (< or ≥70%).

**Methods:**

AF patients with cancer from the nationwide Italian Survey on Anticoagulated Patients Register on oral anticoagulants were included. Propensity score matching (PSM) was performed. Results were expressed as hazard ratio (HR) and 95% confidence interval (CI) for all‐cause mortality and as subdistribution HR (sHR) for CVEs and bleeding risk. Numbers‐needed‐to‐treat (NNT) and numbers‐needed‐to‐harm (NNH) were calculated. VKA patients were stratified by TiTR <70% or ≥70% and compared with DOACs users.

**Results:**

A total of 1605 patients were included (median, 78 years; 44.7% women). During a mean follow‐up of 729.8 days, 153 deaths, 177 CVEs, and 90 bleedings occurred. After PSM, DOACs were associated with lower all‐cause mortality (HR, 0.37, *p* < .001) and CVEs (sHR 0.58, *p* = .005) and similar bleeding risk compared to VKAs. The lowest NNT was observed at 24 months (28.2 for mortality and 37.8 for CVEs), whereas NNH was not significant. DOAC use was associated with lower mortality and CVEs risk in patients with TiTR <70%, and with lower mortality, similar CVEs risk and higher risk of bleeding for a TiTR ≥70%.

**Conclusion:**

DOACs may reduce mortality in AF patients with cancer regardless TiTR. The use of DOACs in high‐bleeding risk patients with good TiTR should be cautious.

## Graphical abstract

Patients with atrial fibrillation (AF) and cancer treated with direct oral anticoagulants (DOACs) had a lower risk of all‐cause mortality, cardiovascular events (CVEs), but not bleeding events, compared to patients treated with vitamin K antagonists (VKAs) with low anticoagulation quality. Conversely, a low all‐cause mortality risk, an increased risk of bleeding, without a clear benefit on CVEs were observed in AF patients with cancer while on DOACs compared to patients on VKAs and high anticoagulation quality.

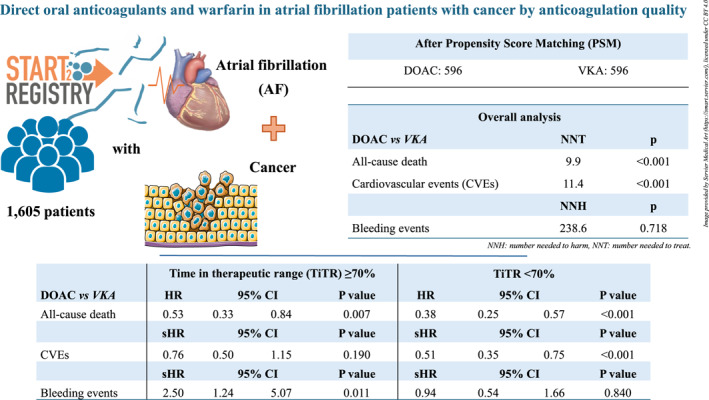

## INTRODUCTION

Atrial fibrillation (AF) and cancer have a bidirectional relationship, sharing common cardiovascular and noncardiovascular risk factors.^1^ The risk of AF varies according to cancer type, stage, and treatments.^2^ The onset of AF may be promoted by the presence of cancer itself^3^ and by cancer treatments such as surgery, chemotherapy, radiotherapy,^4^, ^5^ or pain relief medications such as opioids.^6^, ^7^ In a large cohort of 4,324,545 subjects, the incidence rates of AF resulted consistently higher in patients with cancer compared with the general population (17.4 vs. 3.7 per 1000 person‐years), corresponding to an approximately 5‐fold higher risk of developing AF.^8^ The highest incidence was observed in patients with lung cancer, in whom AF occurred more than 15 times more frequently than in general population.^8^

Furthermore, cancer may promote new‐onset AF due to a direct proinflammatory effect, as well as by inducing metabolic or electrolyte disorders, hypoxemia, or through autonomic dysregulation.^7^ On the other hand, AF patients have an increased risk of developing cancer. Indeed, in patients with newly diagnosed AF, an elevated cancer risk was highest within 1 year (standardized incidence rate [SIR] = 2.30) and persisted beyond 10 years after diagnosis (SIR = 1.18).^9^ This risk increased proportionally according to the presence of risk factors such as age ≥65 years, male gender, hypertension, diabetes, chronic obstructive pulmonary disease (COPD), and liver cirrhosis (hazard ratio [HR] increased from 1.40 for one risk factor to 5.14 for six risk factors).^9^

The concomitant presence of cancer and AF is associated with a higher mortality^10^, ^11^ and thromboembolic and bleeding risk,^12^, ^13^ making the management of anticoagulant therapy with direct oral anticoagulants (DOACs) or vitamin K antagonists (VKAs) challenging in this high‐risk population.

Several studies showed that in patients without cancer, DOACs are associated with better outcomes compared to VKAs, regardless of international normalized ratio (INR) control,^14^ therefore they are recommended as a first line therapeutic option.^15^ However, the role of DOACs in AF patients with cancer^16^ is more controversial with mixed results. Furthermore, there is concern for drug–drug interaction between anticoagulants and antineoplastic drugs.^17^ Therefore, the treatment of patients with AF and cancer is still heterogeneous with some patients still treated with VKAs, low‐molecular weight heparins, or even left untreated.^18^ In addition, previous evidence showed that patients with AF and cancer generally have low quality anticoagulation with VKAs.

One validated parameter to evaluate the quality of anticoagulation during VKA therapy is the time‐in‐therapeutic‐range (TiTR) that represents the time spent within the therapeutic range of INR, which is 2.0–3.0 for patients with AF.

In patients with cancer, the TiTR is particularly low especially in the first 6 months after cancer diagnosis.^19^ This is of concern, as previous evidence showed that a low TiTR (such as <70%) is a good predictor of adverse outcomes for patients while on VKAs.^20^

Likewise, we hypothesized that the effectiveness and safety of DOACs in patients with AF and cancer may be influenced by the TiTR, but to date, no study compared DOACs with VKAs according to the quality of anticoagulation in this clinical setting.

On this basis, we analyzed the risk of all‐cause mortality, cardiovascular events (CVEs), and any bleeding in patients with AF and cancer enrolled within the nationwide Survey on Anticoagulated Patients Register (START) registry according to different anticoagulant treatments.

## MATERIALS AND METHODS

### START registry

The START is a prospective, multicenter, and observational nationwide registry collecting data on AF patients starting anticoagulant therapy in Italy. The START registry has been previously registered on ClinicalTrials.gov (NCT02219984) and its details have been previously described. Only AF patients with active cancer or recent history of cancer (<5 years) were included in the analysis. The enrollment included patients aged ≥18 years with a diagnosis of nonvalvular AF on initiation of anticoagulation with either VKAs or DOACs.

### Ethics statement

The study protocol was accepted by the institutional review board of each participating center, and informed consent was obtained from patients at enrollment. The study protocol complies with the ethical guidelines of the 1975 Helsinki Declaration, and informed consent was obtained from each patient.

### Exclusion criteria

Patients treated with low molecular‐weight heparin were excluded as well as patients already enrolled in phase 2 or 3 clinical studies. Patients enrolled in other observational, or phase 4 studies were considered eligible for the study.

### Patient and public involvement statement

Patients and members of the public were not involved in the design, conduct, reporting, or dissemination plans of this research. They did not contribute to the development of the research question, outcome measures, or study design, nor were they involved in participant recruitment or in assessing the burden of the intervention.

### Baseline characteristics

Patient’s clinical features are recorded by participants on web‐based case report forms (CRF). Baseline data are demographic and clinical characteristics of patients, including cardiovascular risk factors, comorbidities such as peripheral artery disease (PAD), heart failure (HF), chronic kidney disease (CKD) and coronary artery disease (CAD), laboratory routine data, smoking habits, indication for anticoagulant treatment, type of oral anticoagulation, and concomitant drugs. CAD was defined as history of coronary artery disease (either ischemic heart disease or coronary revascularization with stent or coronary artery bypass graft), whereas cerebrovascular disease is defined as previous ischemic stroke or transient ischemic attack. HF was defined as medical history of hospital admission for HF and/or symptoms and signs suggestive for HF. CKD was defined according to an estimated glomerular filtration rate (eGFR) <60 mL/min. Peripheral artery disease was defined according to European Society of Cardiology guidelines.^21^

### Type of anticoagulation

Patients were prescribed DOACs or VKAs at physicians’ discretion. The quality of anticoagulation was expressed by the TiTR that was calculated according to the method of linear interpolation described by Rosendaal et al.^22^ We stratified patients by TiTR for VKA, using a threshold of 70%. Specifically, TiTR stratification was performed only within the VKA cohort, and the same DOAC population was used as the comparator in each stratum (<70% and ≥70%); to further clarify, a schematic illustration of the performed comparisons can be found in Figure S1.

### Definition of cancer

Active cancer or recent history of cancer (<5 years) were included in the analysis. Gastrointestinal, genitourinary, respiratory tract, hematology, breast, and others were used to categorize malignancies of the cohort of our study. Cancer was labeled as “not well specified,” where histological and/or clinical classification was not possible.

### Study end points

The primary end point of the study was the evaluation of all‐cause mortality during follow‐up, comparing patients treated with DOACs to those treated with VKAs. We also evaluated the risk of CVEs, defined as a composite of stroke, systemic embolism, myocardial infarction, and cardiovascular death. We evaluated any bleeding occurred during follow‐up as safety end point, including major bleeding and clinically relevant nonmajor bleeding (CRNMB) as defined by the International Society on Thrombosis and Haemostasis.^23^

### Statistical analysis

Categorical variables were reported as numbers and percentages and were compared using Pearson’s χ^2^ test. Normal distribution of variables was assessed with the Kolmogorov‐Smirnov test. Mean and standard deviation (SD) or median and interquartile range (IQR) were used for continuous variables and were compared by Student’s *t*‐test or Mann‐Whitney *U* test, respectively. Baseline characteristics were reported according to type of anticoagulant administrated (DOAC or VKA).

To address baseline differences between patients treated with VKAs and DOACs, we also performed a propensity score matching (PSM) analysis (summary of balance was reported in Table S1). The propensity score was estimated using logistic regression, incorporating the following covariates: age, sex, paroxysmal AF, CAD, HF, PAD, CKD, alcohol use, dementia, use of a wheelchair, living alone, and family support. Patients were matched 1:1 using nearest‐neighbor matching without replacement, applying a caliper of 0.1. Covariate balance after matching was assessed using standardized mean differences (SMDs), with values <0.15 considered indicative of adequate balance. Balance diagnostics through a Love plot is shown in Figure S2.

Following matching, the cumulative incidence of all‐cause mortality was estimated using a Kaplan–Meier product‐limit estimator. Survival curves were formally compared using the log‐rank test. We evaluated overall mortality risk using a Cox proportional hazards model and results were reported as HR with corresponding 95% confidence intervals (CIs).

We conducted analogous time‐to‐event analyses for CVEs and bleeding outcomes. Cumulative incidence functions (CIFs) were plotted to illustrate event probabilities over time, and Gray’s test was used for between‐group comparisons. Fine–Gray subdistribution hazard models were used to account for the competing risk of all‐cause mortality, and results were reported as subdistribution hazard ratios (sHRs) with corresponding 95% CIs.

To assess consistency of results across different modeling strategies, we also performed univariable and multivariable Cox proportional hazards regression analyses to estimate adjusted HRs and 95% CIs for all‐cause mortality associated with clinically relevant covariates, and, to account for competing risks, both univariable and multivariable Fine–Gray subdistribution hazard models were employed to estimate CVEs and bleeding risk. Similarly to main analysis, results from competing risk analyses were reported as sHRs with corresponding 95% CIs. Additionally, a sensitivity analysis was conducted using a compound exposure variable integrating anticoagulant treatment (DOAC vs. VKA) and VKA TiTR categories.

The number‐needed‐to‐treat (NNT) for all‐cause of death, CVEs and the number‐needed‐to‐harm (NNH) for bleeding events at 6, 12, and 24 months were calculated.

Finally, we performed exploratory subgroup analysis to assess all‐cause mortality, CVEs, and bleeding risk by individual type of DOAC. Bleeding risk evaluation in DOAC patients compared to VKA patients with low or high TiTR was exploratory.

Only *p* values <.05 were considered as statistically significant. All tests were two‐tailed, and analyses were performed using computer software packages (IBM SPSS‐25, SPSS, Inc, R software version 4.2.3, and MedCalc).

## RESULTS

Overall, 1605 patients were finally enrolled with a mean age of 78 years. A total of 44.7% of the patients were women. Summary statistics for matched and unmatched cohorts are shown in Table 1. After PSM, 596 patients treated with VKAs and 596 patients treated with DOACs were analyzed, with all baseline characteristics achieving adequate balance based on SMDs.

**TABLE 1:** Population characteristics according to oral anticoagulants.

	Before matching			After matching		
	VKA, *N* = 684	DOAC, *N* = 921	SMD	VKA, *N* = 596	DOAC, *N* = 596	SMD
Clinical characteristics						
Age (years)	77.00 (71.00, 82.00)	79.00 (74.00, 84.00)	0.2852	78.00 (73.00, 83.00)	78.00 (73.00, 83.00)	0.0054
Female	299 (44%)	423 (46%)	0.0417	269 (45%)	264 (44%)	–0.0168
BMI, median (IQR)	25.70 (23.20–29.10)	25.70 (23.40–28.70)	0.0486	25.90 (23.40– 29.30)	25.60 (23.15– 28.70)	–0.0011
Hypertension	554 (82%)	769 (84%)	0.0611	496 (83%)	500 (84%)	0.0183
Diabetes	143 (21%)	184 (20%)	–0.0251	119 (20%)	122 (20%)	0.0126
CAD	142 (21%)	133 (15%)	–0.1824	105 (18%)	106 (18%)	0.0048
Heart failure	150 (22%)	185 (20%)	–0.048	125 (21%)	114 (19%)	–0.046
Anemia	213 (31%)	294 (32%)	0.0146	187 (31%)	189 (32%)	0.0072
eGFR Median (IQR)	58.00 (42.00–75.00)	59.00 (47.00–75.50)	0.0897	59.00 (43.00–75.50)	59.00 (47.00–74.00)	0.035
Previous stroke/TIA	86 (13%)	144 (16%)	0.0834	74 (12%)	75 (13%)	0.0046
COPD/OSAS	82 (12%)	121 (13%)	0.0329	74 (12%)	83 (14%)	0.0446
Therapy						
Antiplatelet	87 (13%)	90 (9.8%)	–0.101	70 (12%)	71 (12%)	0.0056
Class 1c AAD	49 (7.2%)	99 (11%)	0.1153	48 (8.1%)	60 (10%)	0.0648
Amiodarone	76 (11%)	107 (12%)	0.0147	66 (11%)	63 (11%)	–0.0157
Beta blockers	372 (55%)	404 (44%)	–0.2168	308 (52%)	294 (49%)	–0.0473

Abbreviations: AADs, antiarrhythmic drugs; BMI, body mass index; CAD, coronary artery disease; COPD/OSAS, chronic obstructive pulmonary disease/obstructive sleep apnea syndrome; DOAC, direct oral anticoagulants; eGFR, estimated glomerular filtration rate; IQR, interquartile range; SMD, standardized mean difference; TIA, transient ischemic attack; VKA, vitamin K antagonist.

Overall, 292 (18.2%) patients had active cancer at the time of enrollment. The most common cancer types were genitourinary (27.6%), gastrointestinal (16.4%), breast (18.2%), hematological (9.3%), respiratory tract (3.0%), and others (8.5%). Cancer site was not specified in 14.5%, and 2.5% of patients had more than one cancer (Table S2).

### DOACs and all‐cause mortality

**FIGURE 1:**
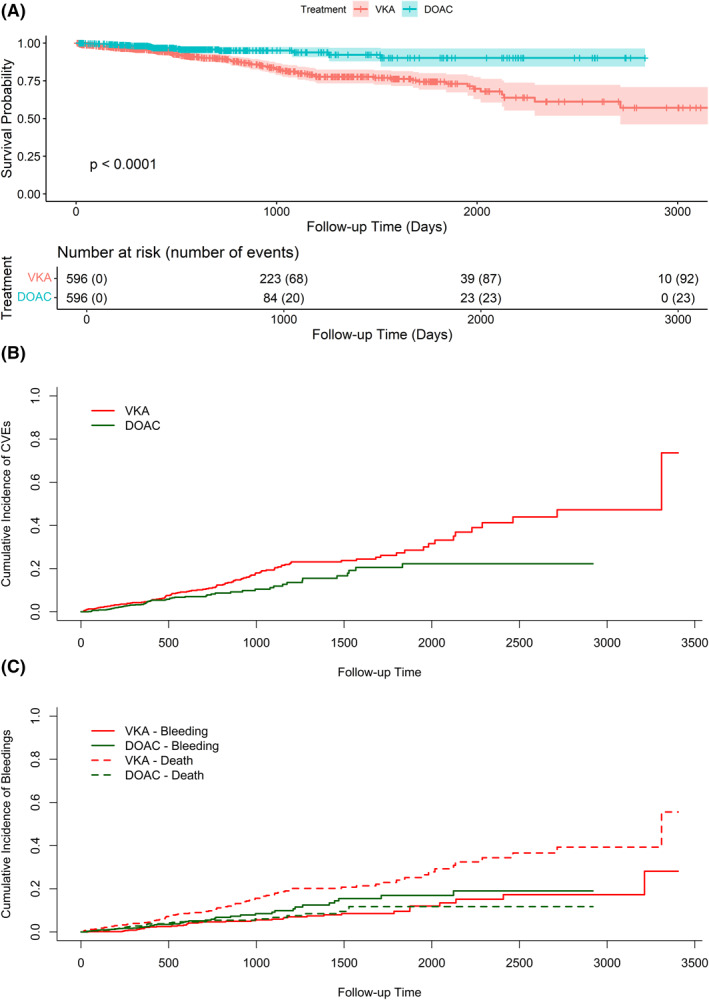
Kaplan–Meier survival curves (A) and cumulative incidence function for cardiovascular events (B) and for bleeding events (C) according to anticoagulant treatment. DOAC indicates direct oral anticoagulant; VKA, vitamin K antagonist.

During a mean follow‐up of 729.8 ± 597.4 days, a total of 153 deaths occurred (4.77 per 100 patient‐years). Kaplan–Meier survival curves (Figure 1A) showed a lower incidence of deaths in patients treated with DOACs compared to VKAs (log‐rank test *p* < .001).

At univariable Cox regression analysis (Table 2, panel A) for all‐cause mortality performed after PSM, DOACs were associated with lower all‐cause mortality risk (HR, 0.37; 95% CI, 0.23–0.58, *p* < .001).

**TABLE 2:** Univariable Cox regression analyses for all‐cause mortality (panel A) and Fine–Gray univariable analysis for cardiovascular (panel B) and bleeding events (panel C) according to anticoagulant treatment.

	HR	95% CI	*p*
Panel A			
DOAC vs. VKA	0.37	0.23–0.58	<.001

	**sHR**	**95% CI**	***p***
Panel B			
DOAC vs. VKA	0.58	0.39–0.84	.005

	**sHR**	**95% CI**	***p***
Panel C			
DOAC vs. VKA	1.38	0.84–2.28	.210

Abbreviations: DOAC, direct oral anticoagulants; HR, hazard ratio; sHR, subdistributional hazard ratio; VKA, vitamin K antagonists.

Multivariable Cox regression analysis performed on 1605 AF patients and adjusted for all clinical baseline variables confirmed these results (DOACs vs. VKA, HR, 0.47; 95% CI, 0.33–0.68; *p* < .001).

### DOACs and CVEs

During the follow‐up, 177 CVEs were observed (5.52 per 100 patient‐years). CIF curves (Figure 1B) showed a lower incidence of CVEs in patients treated with DOACs without competitive event risk.

At univariable Fine–Gray analysis (Table 2, panel B) for CVEs performed after PSM, DOACs were associated with lower CVEs risk (HR, 0.58; 95% CI, 0.39–0.84, *p* = 0.005).

Multivariable Fine–Gray analysis adjusted for all clinical baseline variables confirmed these results (DOAC vs. VKA, HR, 0.67; 95% CI, 0.48–0.92; *p* = .013). Full multivariable model is reported in Table S3.

### DOAC and bleeding events

During follow‐up, 90 bleeding events occurred (2.80 per 100 patient‐years), specifically 40 major bleedings and 50 CRNMBs. CIF curves (Figure 1C) showed a lower incidence of bleeding in patients treated with DOACs.

Univariable Fine–Gray model (Table 2, panel C) performed after PSM showed that the use of DOACs was not significantly associated with a higher risk of bleeding (sHR, 1.38; 95% CI, 0.84–2.28, *p* = .210).

Multivariable Fine–Gray analysis adjusted for all clinical baseline variables confirmed these results (DOAC vs. VKA, HR, 1.42; 95% CI, 0.88–2.28; *p* = .150). Full multivariable model is reported in Table S3.

### NNT and NNH

We calculated the NNT and NNH at 6, 12, and 24 months of follow‐up (Table 3). Overall, DOAC use had a favorable NNT for all‐cause mortality and CVEs without a significant NNH for any bleeding (Table 3).

**TABLE 3:** NNT for all‐cause mortality risk and cardiovascular events and NNH for bleeding events according to anticoagulant treatment.

DOAC vs. VKA	Overall		6 months		12 months		24 months	
	NNT	*p*	NNT	*p*	NNT	*p*	NNT	*p*
All‐cause mortality	9.9	<.001	75.4	.057	125.8	.391	28.2	.003
CVEs	11.4	<.001	79.6	.084	138.1	.447	37.8	.037

DOAC vs. VKA	Overall		6 months		12 months		24 months	
	NNT	*p*	NNT	*p*	NNT	*p*	NNT	*p*
Bleeding events	238.6	.718	95.3	.044	116.5	.207	300.2	.721

Abbreviations: CI, confidence interval; CVEs, cardiovascular events; NNH, number‐needed‐to‐harm; NNT, number‐needed‐to‐treat.

The NNT for DOAC decreased significantly according to the length of follow‐up, suggesting a more favorable association of DOACs with clinical outcomes over time (Table 3). NNH was not different between DOACs and VKA at 6, 12, and 24 months of follow‐up and always exceeded the NNT (Table 3).

### Anticoagulation quality

Among VKA‐treated patients, the mean TiTR of VKA therapy was 64.3% (±19.4), and 31.7% of patients had a TiTR ≥70%. When compared with VKA patients with TiTR <70%, DOACs use was associated with a lower risk of all‐cause mortality and CVEs, with similar risk of bleeding at univariable and multivariable analysis (Table 4).

**TABLE 4:** Univariable and multivariable Cox regression analysis of factors associated with all‐cause death (panel A) and univariable and multivariable Fine–Gray analysis of cardiovascular events (panel B) and bleedings (panel C) according to TiTR above and under 70%.

TiTR <70%	HR	95% CI	*p*
Panel A			
DOAC vs. VKA^a^	0.34	0.23–0.51	<.001
DOAC vs. VKA^b^	0.38	0.25–0.57	<.001

TiTR <70%	**sHR**	**95% CI**	***p***
Panel B			
DOAC vs. VKA^a^	0.47	0.33–0.68	<.001
DOAC vs. VKA^b^	0.51	0.35–0.75	<.001
Panel C			
DOAC vs. VKA^a^	0.87	0.53–1.44	.590
DOAC vs. VKA^b^	0.94	0.54–1.66	.840

TiTR ≥70%	HR	95% CI	*p*
Panel A			
DOAC vs. VKA^a^	0.57	0.36–0.89	.013
DOAC vs. VKA^b^	0.53	0.33–0.84	.007

TiTR <70%	**sHR**	**95% CI**	***p***
Panel B			
DOAC vs. VKA^a^	0.81	0.54–1.21	.310
DOAC vs. VKA^b^	0.76	0.50–1.15	.190
Panel C			
DOAC vs. VKA^a^	2.56	1.29–5.07	.007
DOAC vs. VKA^b^	2.50	1.24–5.07	.011

Abbreviations: CI, confidence interval; DOAC, direct oral anticoagulants; HR, hazard ratio; sHR, subdistribution hazard ratio; TiTR, time in therapeutic range; VKA, vitamin K antagonist.

^a^ Univariable.

^b^ Multivariable (adjusted for all clinical baseline characteristics).

When compared with VKA patients with TiTR ≥70%, DOACs use remained associated with a lower risk of all‐cause mortality and similar risk of CVEs, but with a higher risk of any bleeding in both univariable and multivariable analyses (Table 4).

All these findings were consistent in sensitivity analyses using a compound exposure approach combining anticoagulant treatment and VKA TiTR categories, which showed comparable effect estimates across outcomes (Table S4).

### Type of DOACs

**FIGURE 2:**
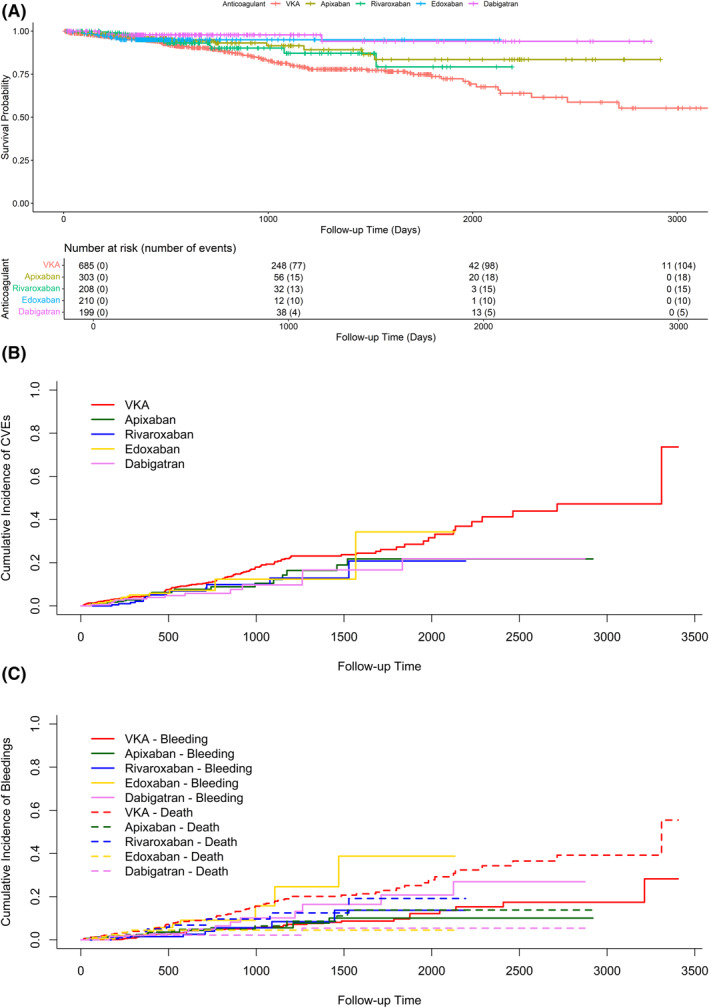
Kaplan–Meier survival curves (A) and cumulative incidence function for cardiovascular events (B) and for bleeding events (C) according to each type of anticoagulant. Dashed line indicates deaths; solid line, bleeding; VKA, vitamin K antagonist.

We observed a significant difference at Kaplan–Meier curves for all‐cause mortality (Figure 2A), and at CIF curves for CVEs (Figure 2B) but not for bleedings (Figure 2C) between patients treated with DOACs or VKAs. Apixaban and dabigatran were associated with lower risk of all‐cause mortality compared to VKAs at univariable Cox regression analysis, whereas no significant differences were observed between rivaroxaban, edoxaban, and VKAs (Table 5, panel A). At Fine–Gray analysis, only dabigatran showed a lower risk of CVEs compared to VKA (HR, 0.54; 95% CI, 0.31–0.95, *p* = .031) (Table 5, panel B), whereas only edoxaban was associated with a higher risk of any bleeding compared to VKAs (HR, 2.41; 95% CI, 1.27–4.57, *p* = .007) (Table 5, panel C).

**TABLE 5:** Univariable Cox regression analysis of factors associated with all‐cause death (panel A), cardiovascular events (panel B), and bleedings (panel C) according to each direct oral anticoagulant.

	HR	95% CI low–high	*p*
Panel A			
Apixaban vs. VKA	0.52	0.31–0.85	.010
Rivaroxaban vs. VKA	0.67	0.39–1.16	.154
Edoxaban vs. VKA	0.60	0.31–1.15	.124
Dabigatran vs. VKA	0.20	0.08–0.49	<.001

	**sHR**	**95% CI low–high**	***p***
Panel B			
Apixaban vs. VKA	0.67	0.43–1.05	.078
Rivaroxaban vs. VKA	0.66	0.38–1.12	.120
Edoxaban vs. VKA	0.83	0.47–1.46	.520
Dabigatran vs. VKA	0.54	0.31–0.95	.031
Panel C			
Apixaban vs. VKA	1.04	0.55–1.96	.910
Rivaroxaban vs. VKA	0.97	0.45–2.08	.930
Edoxaban vs. VKA	2.41	1.27–4.57	.007
Dabigatran vs. VKA	1.59	0.86–2.94	.140

Abbreviations: CI, confidence interval; HR, hazard ratio; sHR, subdistribution hazard ratio; VKA, vitamin K antagonists.

An exploratory comparison of single DOACs and VKA with TiTR ≥70%, at univariable analysis, edoxaban, and dabigatran (HR, 4.37; 95% CI, 1.86–10.29, *p* = .001 and HR, 2.59; 95% CI, 1.14–5.88, *p* = .023, respectively) but not apixaban and rivaroxaban (HR, 1.73; 95% CI, 0.75–3.98, *p* = .196 and HR, 1.69; 95% CI, 0.66–4.35, *p* = .276, respectively) were associated with higher risk of bleeding. These results were confirmed in a multivariable analysis adjusted for CHA_2_DS_2_‐VASc (HR, 4.36; 95% CI, 1.86–10.29, *p* = .001 for edoxaban and HR, 2.59; 95% CI, 1.14–5.88, *p* = .023 for dabigatran).

## DISCUSSION

In our study, the comparison of DOACs with VKAs according to anticoagulation quality showed that compared with patients in VKA group with low TiTR, DOACs use was associated with lower all‐cause mortality and CVEs, and similar bleeding risk. Conversely, compared to patients receiving VKA with a TiTR ≥70%, DOAC use was associated with a lower all‐cause mortality risk, but without a clinical benefit on CVEs, and with an increased bleeding risk compared to VKA. These findings should be interpreted as reflecting differences in the quality of VKA anticoagulation used as the comparator. To better understand this result, we performed an exploratory analysis on the association of each DOAC with bleeding, and we found a higher bleeding risk in patients treated with edoxaban or dabigatran.

In patients on VKAs in our study, the mean TiTR was 64.3%. Few previous studies reported data on anticoagulation quality in patients with AF and cancer. In the Rivaroxaban Once Daily Oral Direct Factor Xa Inhibition Compared with Vitamin K Antagonism for Prevention of Stroke and Embolism Trial in Atrial Fibrillation trial,^24^ the median TiTR for patients taking warfarin was 58% in those without a history of cancer and 64% in those with a history of cancer. A similar TiTR between patients with and without cancer was found in the warfarin arm in the Effective Anticoagulation with Factor Xa Next Generation in Atrial Fibrillation–Thrombolysis in Myocardial Infarction 48 trial^25^ (median TiTR, 68.2% vs. 68.4%, respectively).

The TiTR is not reported according to cancer status in the Apixaban for Reduction in Stroke and Other Thromboembolic Events in Atrial Fibrillation trial, and no post hoc analysis from the Randomized Evaluation of Long‐Term Anticoagulation Therapy trial on this topic has been performed.

However, the good value of TiTR observed in clinical trials may be influenced by the controlled setting of the clinical studies. Indeed, in observational real‐world studies, several did not even report data on TiTR,^26^, ^27^, ^28^, ^29^, ^30^ whereas for those with available data, evidence showed a lower median TiTR ranging from 23.9 to 59.5%.^31^, ^32^

In the ORBIT‐AF registry,^33^ a similar TiTR in patients with and without cancer was observed (median TiTR 68% vs. 67%, respectively, *p* = .10), at the expense of a greater number of INR checks (28 vs. 24 checks, *p* < .0001) and with a higher proportion of cancer patients was followed by specialized centers for INR monitoring (48.4% vs. 42.6%, *p* < .0001).^33^

In a PSM retrospective cohort of 195 couples of AF patients with active cancer reported no significant differences in bleeding or cerebrovascular events between DOACs and VKAs, but no data on anticoagulation quality in the warfarin arm were reported.^34^

In the retrospective observational Anticoagulants for Reduction in Stroke: Observational Pooled Analysis on Health Outcomes and Experience of Patients study on 40,271 patients with AF and cancer, a lower risk of stroke/SE and major bleeding in patients treated with apixaban was found, whereas other DOACs were not associated with outcomes.^35^ The risk of bleeding is different among DOACs, but no data on edoxaban was available as well as data on VKAs therapy quality.

A new finding of our study comes from the analysis stratified by the quality of VKA anticoagulation, expressed by TiTR. In patients with TiTR <70%, a lower risk of all‐cause mortality and CVEs was associated with DOAC use without a significant increase in bleeding. This suggests that DOACs may represent an optimal choice in AF patients with a suboptimal anticoagulation quality on VKA, as frequently observed in cancer patients.

On the other hand, in patients with TiTR ≥70%, results were more heterogeneous. We confirmed a survival benefit in patients on DOACs, but the incidence of CVEs was similar between the two groups and a higher bleeding risk was found in patients treated with DOACs.

Among DOACs, we found that edoxaban and dabigatran were associated with increased bleeding risk when compared to patients with well‐managed VKA (i.e., TiTR ≥70%). Because this is an exploratory analysis, this result needs to be confirmed by larger studies to have more robust evidence.

Our study has clinical implications. Our findings indicate that quality of VKA therapy, as assessed by the TiTR, is a key factor to evaluate the benefit of DOAC therapy in AF cancer patients. Patients with high‐quality VKA anticoagulation may have a reasonable safety bleeding profile that should be considered before deciding a switch to DOACs.

The observed association between the use of DOACs and the lower all‐cause mortality could be multifactorial, and the exact mechanisms underlying this beneficial association remain to be investigated. This represents a clinical benefit that goes beyond thromboembolism prevention, and for clinicians, these data suggest that anticoagulant choice should be considered as an additional determinant of survival, being part of the global evaluation of patients with AF and cancer. This aspect was confirmed by the low NNT and the nonsignificant NNH obtained in our study, especially during long follow‐up; these data suggest a real benefit from DOACs in reducing CVEs and all‐cause mortality, especially in patients with a mid‐long‐term life expectancy. Notably, because our analysis considered only major bleeding and CRNMBs (excluding minor bleeding), the high NNH for bleeding is not only substantial in absolute terms, but also carries greater clinical relevance.

### Strengths

Strength of our study was represented by the rigorous methodology that confirmed the results using two different types of analysis: PSM and the multivariable analysis and evaluating also the competitive risks, that is considered appropriate for studies including cancer patients.^36^ In addition, the real‐world population enrolled and the long‐term follow‐up in a frail population (such as patients with AF and cancer), represented a further strength of our study. Finally, the prospective multicenter design of the START registry permitted a well characterization of population enrolled, including several comorbidities information collected at baseline compared to retrospective studies that often were subjected to limited clinical information and selection bias.

### Limitations

Our study has limitations. As in all observational studies, residual confounding factors cannot be excluded. In particular, despite the use of PSM, residual confounding by indication cannot be excluded. The START registry does not provide granular information on cancer stage, degree of cancer activity beyond the available binary definition, or details on anticancer treatments. These factors are known to strongly influence prognosis and may have affected treatment allocation and outcomes. Therefore, the observed mortality reduction should be interpreted with caution, as it may not be fully attributable to the anticoagulant effect alone but could partly reflect selection bias and unmeasured confounding related to the underlying malignancy. Moreover, the exploratory comparisons between DOACs and VKAs according to TiTR regarding bleeding events were based on analyses in which the same DOAC cohort was compared with two different VKA subgroups defined by anticoagulation quality. As a result, these comparisons are not statistically independent, and the corresponding HRs should be interpreted with caution. Finally, the cohort lacks ethnic diversity, which may restrict the applicability of our results to other ethnicities.

In conclusion, DOACs seem to have a favorable risk–benefit profile compared to VKAs, with lower risk of all‐cause mortality and CVEs. These differences were more pronounced when DOACs were compared with VKA therapy of lower anticoagulation quality. Overall, the quality of anticoagulation achieved during VKA therapy should be considered when managing anticoagulation strategies in patients with AF and cancer.

## AUTHOR CONTRIBUTIONS

**Danilo Menichelli**: Conceptualization; formal analysis; and writing—original draft. **Vito Maria Daniele Cormaci**: Conceptualization; formal analysis; and writing—original draft. **Gianluca Gazzaniga**: Formal analysis; methodology; and writing—original draft. **Irma Bisceglia**: Visualization; validation; and writing—original draft. **Massimiliano Camilli**: Visualization and validation. **Daniela Poli**: Visualization; data curation; and investigation. **Emilia Antonucci**: Visualization; data curation; and investigation. **Roberto Pola**: Visualization and validation. **Paolo Santini**: Visualization and validation. **Pasquale Pignatelli**: Visualization; validation; writing—review and editing; and supervision. **Daniele Pastori**: Visualization; validation; writing—review and editing; and supervision.

## CONFLICT OF INTEREST STATEMENT

The authors declare no conflicts of interest.

## Supporting information

Supporting Information S1

Supporting Information S2

## Data Availability

The data that support the findings of this study are available on request from the corresponding author. The data are not publicly available due to privacy or ethical restrictions.
