# Supplementary material for: Direct oral anticoagulants and warfarin in atrial fibrillation patients with cancer by anticoagulation quality
Source: Cancer. 2026 Jul 7;132(14):e70501. doi: 10.1002/cncr.70501 (PMC13338909; doi:10.1002/cncr.70501)

**Direct oral anticoagulants and warfarin in atrial fibrillation patients with cancer by anticoagulation quality.**

*Supplementary material*

**Tables**

**Supplementary Table 1. Summary of Balance - All data**

**Supplementary Table 2. Population characteristics according to oral anticoagulants.**

**Supplementary Table 3. Multivariable Fine-Gray Analyses for Cardiovascular Events and Bleedings.**

**Supplementary Table 4. Univariable Cox and Fine-Gray regression analyses of clinical outcomes according to compound exposure combining anticoagulant treatment and VKA time in therapeutic range.**

**Figures**

**Supplementary Figure 1. Schematic illustration of stratification for TiTR classes and comparisons performed**

**Supplementary Figure 2. Love Plot**

**Supplementary Table 1. Summary of Balance - All data**

| **Variable** | **Means Treated** | **Means Control** | **Std. Mean Diff** | **Var. Ratio** | **eCDF Mean** | **eCDF Max** |
| --- | --- | --- | --- | --- | --- | --- |
| distance | 0.6026 | 0.5369 | 0.576 | 0.7519 | 0.1473 | 0.221 |
| Heart failure | 0.202 | 0.2212 | -0.048 |  | 0.0193 | 0.0193 |
| Female | 0.4618 | 0.441 | 0.0417 |  | 0.0208 | 0.0208 |
| Age (years) | 78.5764 | 76.3702 | 0.2852 | 0.8952 | 0.0437 | 0.1166 |
| BMI | 26.4321 | 26.2006 | 0.0486 | 1.0539 | 0.0098 | 0.0408 |
| Hypertension | 0.8395 | 0.8171 | 0.0611 |  | 0.0224 | 0.0224 |
| Diabetes | 0.2009 | 0.2109 | -0.0251 |  | 0.01 | 0.01 |
| CAD | 0.1452 | 0.2094 | -0.1824 |  | 0.0642 | 0.0642 |
| Anaemia | 0.321 | 0.3142 | 0.0146 |  | 0.0068 | 0.0068 |
| eGFR | 63.3603 | 61.1785 | 0.0897 | 0.886 | 0.0207 | 0.0865 |
| Previous stroke/TIA | 0.1572 | 0.1268 | 0.0834 |  | 0.0304 | 0.0304 |
| COPD/OSAS | 0.1321 | 0.1209 | 0.0329 |  | 0.0112 | 0.0112 |
| Antiplatelet | 0.0983 | 0.1283 | -0.101 |  | 0.0301 | 0.0301 |
| Class 1c AAD | 0.1081 | 0.0723 | 0.1153 |  | 0.0358 | 0.0358 |
| Amiodarone | 0.1168 | 0.1121 | 0.0147 |  | 0.0047 | 0.0047 |
| Beta blockers | 0.441 | 0.5487 | -0.2168 |  | 0.1076 | 0.1076 |

Summary of Balance – Matched Data

| **Variable** | **Means Treated** | **Means Control** | **Std. Mean Diff** | **Var. Ratio** | **eCDF Mean** | **eCDF Max** | **Std. Pair Dist.** |
| --- | --- | --- | --- | --- | --- | --- | --- |
| distance | 0.5713 | 0.5627 | 0.0761 | 1.058 | 0.0232 | 0.0587 | 0.0771 |
| Heart failure | 0.1913 | 0.2097 | -0.046 |  | 0.0185 | 0.0185 | 0.7982 |
| Female | 0.443 | 0.4513 | -0.0168 |  | 0.0084 | 0.0084 | 0.9726 |
| Age (years) | 77.4581 | 77.4161 | 0.0054 | 1.1231 | 0.0073 | 0.0235 | 0.9689 |
| BMI | 26.282 | 26.2874 | -0.0011 | 1.1287 | 0.0113 | 0.0386 | 1.0103 |
| Hypertension | 0.8389 | 0.8322 | 0.0183 |  | 0.0067 | 0.0067 | 0.7588 |
| Diabetes | 0.2047 | 0.1997 | 0.0126 |  | 0.005 | 0.005 | 0.8334 |
| CAD | 0.1779 | 0.1762 | 0.0048 |  | 0.0017 | 0.0017 | 0.7954 |
| Anaemia | 0.3171 | 0.3138 | 0.0072 |  | 0.0034 | 0.0034 | 0.8913 |
| eGFR | 62.5201 | 61.6695 | 0.035 | 0.7949 | 0.0235 | 0.0906 | 1.0567 |
| Previous stroke/TIA | 0.1258 | 0.1242 | 0.0046 |  | 0.0017 | 0.0017 | 0.5485 |
| COPD/OSAS | 0.1393 | 0.1242 | 0.0446 |  | 0.0151 | 0.0151 | 0.669 |
| Antiplatelet | 0.1191 | 0.1174 | 0.0056 |  | 0.0017 | 0.0017 | 0.7046 |
| Class 1c AAD | 0.1007 | 0.0805 | 0.0648 |  | 0.0201 | 0.0201 | 0.4864 |
| Amiodarone | 0.1057 | 0.1107 | -0.0157 |  | 0.005 | 0.005 | 0.6634 |
| Beta blockers | 0.4933 | 0.5168 | -0.0473 |  | 0.0235 | 0.0235 | 0.8313 |

**Supplementary Table 2. Population characteristics according to oral anticoagulants.**

|  | Total Cohort  (n:1605) | VKA  (n:684) | DOAC  (n:921) | p-value |
| --- | --- | --- | --- | --- |
| *Age (year)* | 77.7±8.0 | 76.4±8.2 | 78.6±7.7 | <0.001 |
| *Women (%)* | 727 (45.3) | 302 (44.2) | 425 (46.1) | 0.428 |
| *Arterial Hypertension (%)* | 1,333 (83.1) | 560 (81.9) | 773 (83.9) | 0.277 |
| *Diabetes Mellitus (%)* | 328 (20.4) | 143 (20.9) | 185 (20.1) | 0.687 |
| *Cardiovascular Diseases (%)* | 281 (17.5) | 146 (21.3) | 135 (14.7) | <0.001 |
| *Obesity (%)* | 308 (19.2) | 132 (19.3) | 176 (19.1) | 0.924 |
| *Anaemia (%)* | 511 (31.8) | 215 (31.4) | 296 (32.1) | 0.764 |
| *eGFR> 60 mL/min (%)* | 762 (47.6) | 323 (47.3) | 439 (47.8) | 0.850 |
| *eGFR (mL/min)* | 62.4±25.0 | 61.1±25.8 | 63.4±24.4 | 0.071 |
| *Paroxysmal Atrial Fibrillation (%)* | 624 (39.2) | 243 (36.1) | 381 (41.5) | 0.031 |
| *Reduced Ejection Fraction (%)* | 151 (9.7) | 75 (11.1) | 76 (8.6) | 0.095 |
| *Heart Failure (%)* | 341 (21.2) | 154 (22.5) | 187 (20.3) | 0.284 |
| *PAD (%)* | 96 (6.0) | 47 (6.9) | 49 (5.3) | 0.195 |
| *COPD (%)* | 207 (12.9) | 85 (12.4) | 122 (13.2) | 0.628 |
| *HAS-BLED (mean)* | 2.1±0.8 | 2.1±0.8 | 2.1±0.7 | 0.569 |
| *CHA_2_DS_2_-VASc (mean)* | 3.8±1.4 | 3.7±1.4 | 3.8±1.4 | 0.059 |
| Site of cancer | | | | |
| *GI cancer (%)* | 264 (16.4) | 89 (13.0) | 175 (19.0) | <0.001 |
| *GU cancer (%)* | 443 (27.6) | 184 (26.9) | 259 (28.1) |  |
| *Respiratory Tract cancer (%)* | 48 (3.0) | 19 (2.8) | 29 (3.1) |  |
| *Hematological cancer (%)* | 149 (9.3) | 78 (11.4) | 71 (7.7) |  |
| *Breast cancer (%)* | 292 (18.2) | 117 (17.1) | 175 (19.0) |  |
| *Other types of cancer (%)* | 137 (8.5) | 72 (10.5) | 65 (7.1) |  |
| *Two or more cancers (%)* | 40 (2.5) | 24 (3.5) | 16 (1.7) |  |
| *Not specified (%)* | 232 (14.5) | 101 (14.8) | 131 (14.2) |  |
| *Active cancer (%)* | 292 (18.2) | 126 (18.4) | 166 (18.0) | 0.926 |
| Therapy | | | | |
| *Antiplatelet drugs (%)* | 177 (11.0) | 87 (12.7) | 90 (9.8) | 0.062 |
| *Class I C Antiarrhythmics (%)* | 149 (9.3) | 49 (7.2) | 100 (10.9) | 0.013 |
| *Amiodarone (%)* | 183(11.4) | 76 (11.1) | 107 (11.6) | 0.752 |
| *Lipid Lowering Drug Therapy (%)* | 519 (32.3) | 237 (34.6) | 282 (30.6) | 0.088 |
| *RAAS-i (%)* | 905 (56.4) | 387 (56.6) | 518 (56.2) | 0.893 |
| *Beta- Blockers (%)* | 781 (48.7) | 376 (55.0) | 405 (44.0) | <0.001 |
| *Calcium channel blockers (%)* | 376 (23.4) | 164 (24.0) | 212 (23.0) | 0.654 |
| *Diuretics (%)* | 616 (38.4) | 273 (39.9) | 343 (37.2) | 0.277 |
| *Digoxin (%)* | 122 (7.6) | 58 (8.5) | 64 (6.9) | 0.253 |
| *PPI (%)* | 588 (36.6) | 272 (39.8) | 316 (34.3) | 0.025 |

*COPD: Chronic Obstructive Pulmonary Disease, eGFR: Estimated Glomerular Filtration Rate, GI: gastrointestinal, GU: genitourinary, PAD: Peripheral Artery Disease, PPI: Proton Pump Inhibitors, RAAS-i: Renin Angiotensin Aldosterone System inhibitors, TiTR: time in therapeutic range.*

**Supplementary Table 3. Multivariable Fine-Gray Analyses for Cardiovascular Events and Bleedings.**

|  | Cardiovascular Events | | | Bleeding | | |
| --- | --- | --- | --- | --- | --- | --- |
|  | **sHR** | **95% CI** | **p-value** | **sHR** | **95% CI** | **p-value** |
| *DOAC (vs VKA)* | 0.67 | 0.48 - 0.92 | 0.013 | 1.42 | 0.88 - 2.28 | 0.150 |
| *Age (years)* | 1.03 | 1.01 - 1.06 | 0.016 | 1.00 | 0.97 - 1.03 | 0.920 |
| *Female* | 0.72 | 0.52 - 0.99 | 0.045 | 0.76 | 0.48 - 1.20 | 0.240 |
| *BMI* | 1.04 | 1.01 - 1.07 | 0.023 | 1.01 | 0.97 - 1.06 | 0.550 |
| *eGFR* | 0.99 | 0.98 - 1.00 | 0.003 | 1.00 | 0.99 - 1.01 | 0.850 |
| *Hypertension* | 0.97 | 0.64 - 1.47 | 0.880 | 1.02 | 0.55 - 1.90 | 0.940 |
| *Diabetes* | 0.83 | 0.56 - 1.21 | 0.330 | 1.08 | 0.65 - 1.81 | 0.760 |
| *CAD* | 1.4 | 0.98 - 2.00 | 0.064 | 1.39 | 0.81 - 2.39 | 0.230 |
| *Anaemia* | 1.4 | 1.02 - 1.91 | 0.035 | 1.72 | 1.11 - 2.67 | 0.016 |
| *Heart failure* | 1.08 | 0.74 - 1.58 | 0.700 | 0.85 | 0.49 - 1.50 | 0.580 |
| *PAD* | 1.62 | 1.07 - 2.47 | 0.023 | 0.65 | 0.24 - 1.77 | 0.400 |
| *COPD/OSAS* | 1.29 | 0.87 - 1.91 | 0.200 | 1.56 | 0.88 - 2.75 | 0.130 |

*BMI: body mass index; CAD: coronary artery disease; CI: Confidence Interval; COPD/OSAS: chronic obstructive pulmonary disease/obstructive sleep apnoea syndrome; DOAC: direct oral anticoagulants; eGFR: estimate glomerular filtration rate; PAD: peripheral artery disease; sHR: subdistribution Hazard Ratio; VKA: vitamin K antagonist.*

**Supplementary Table 4. Univariable Cox and Fine-Gray regression analyses of clinical outcomes according to compound exposure combining anticoagulant treatment and Vitamin K antagonist time in therapeutic range.**

| ***VKA vs DOAC*** | | | |
| --- | --- | --- | --- |
| **All-cause mortality** | **HR** | **95% CI** | **p-value** |
| TiTR <70% | 2.86 | 1.91, 4.28 | <0.001 |
| TiTR ≥70% | 1.67 | 1.07, 2.61 | 0.024 |
| **CVE** | **sHR** | **95% CI** | **p-value** |
| TiTR <70% | 2.08 | 1.45, 3.00 | <0.001 |
| TiTR ≥70% | 1.19 | 0.79, 1.79 | 0.400 |
| **Bleeding** | **sHR** | **95% CI** | **p-value** |
| TiTR <70% | 1.15 | 0.69, 1.89 | 0.590 |
| TiTR ≥70% | 0.42 | 0.22, 0.82 | 0.011 |

*Reference Category: DOAC.
Abbreviations: CI: Confidence Interval; DOAC: direct oral anticoagulants, HR: Hazard Ratio; sHR: Subdistribution Hazard Ratio; TiTR: Time in Therapeutic Range, VKA: vitamin K antagonist.*

**Supplementary Figure 1. Schematic illustration of stratification for TiTR classes and comparisons performed.**

**
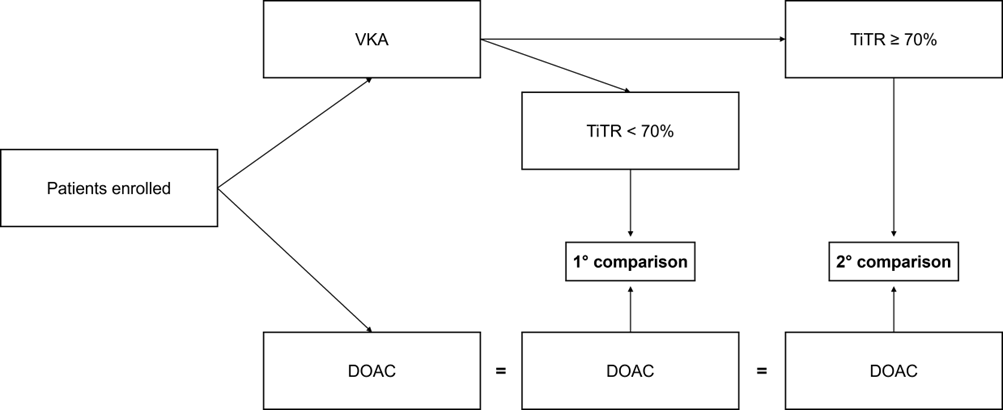
**

**Supplementary Figure 2. Love Plot**


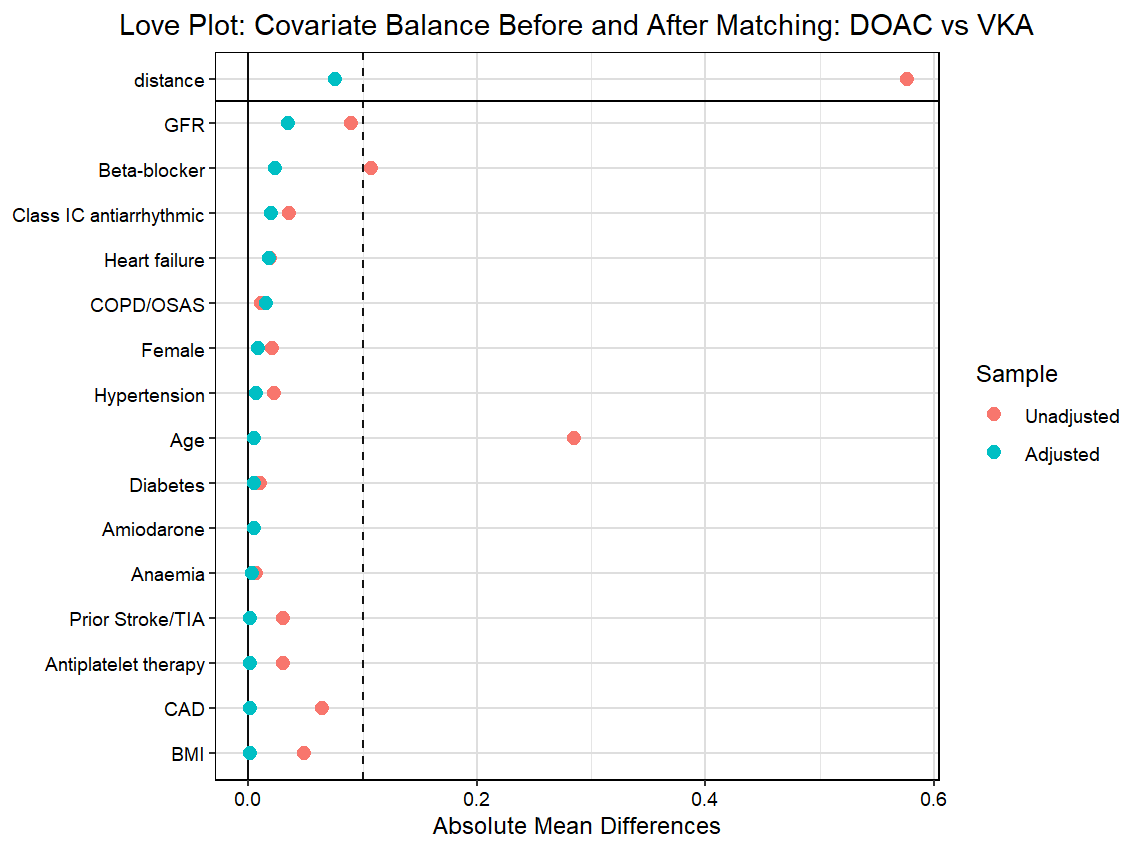

Supplement: Supplementary file 1 — Supporting Information S1 [file CNCR-132-e70501-s002.docx]
