# Supplementary material for: Direct oral anticoagulants and warfarin in atrial fibrillation patients with cancer by anticoagulation quality
Source: Cancer. 2026 Jul 7;132(14):e70501. doi: 10.1002/cncr.70501 (PMC13338909; doi:10.1002/cncr.70501)
Supplement: Supplementary file 2 — Supporting Information S2 [file CNCR-132-e70501-s001.docx]

**Direct oral anticoagulants and warfarin in atrial fibrillation patients with cancer by anticoagulation quality.**

*Short title: DOACs in AF with cancer: mortality and CVEs.*

**Appendix**

**START2 Register Investigators.**

**Sophie Testa**, Oriana Paoletti, UO Laboratorio Analisi, Centro Emostasi e Trombosi A O Istituti Ospitalieri di Cremona, Cremona; **Benilde Cosmi, Giuliana Guazzaloca, Ludovica Migliaccio**, UO di Angiologia e Malattie Coagulazione, AOU S. Orsola-Malpighi, Bologna, Bologna; **Daniela Poli, Rossella Marcucci, Niccolò Maggini**, SOD Malattie Aterotrombotiche, Azienda Ospedaliero Universitaria-Careggi, Firenze; **Vittorio Pengo**, Dipartimento di Scienze Cardio-Toraco-Vascolari, Centro Trombosi, AOU Padova, Padova; **Anna Falanga, Teresa Lerede**, USC SIMT, Centro Emostasi e Trombosi , Ospedale Papa Giovanni XXIII, Bergamo; **Lucia Ruocco**, U.O. Analisi Chimico-Cliniche, Azienda Ospedaliero Universitaria Pisana, Pisa; **Giuliana Martini**, Centro Emostasi, Spedali Civili Di Brescia, Brescia; **Simona Pedrini, Federica Bertola**, Servizio di Laboratorio, Istituto Ospedaliero Fondazione Poliambulanza Brescia; **Lucilla Masciocco, Pasquale Saracino, Angelo Benvenuto**, UOC Medicina Interna, Centro Controllo Coagulazione, Presidio Ospedaliero Lastaria, Lucera (Foggia); **Claudio Vasselli**, Laboratorio Patologia Clinica, Policlinico Casilino, Roma; **Francesco Violi,** **Pasquale Pignatelli, Daniele Pastori** Centro Trombosi, Clinica Medica Policlinico Umberto I°, Università la Sapienza Roma; **Elvira Grandone, Donatella Colaizzo** Centro Trombosi Casa del Sollievo e della Sofferenza, S.Giovanni Rotondo (Foggia); **Marco Marzolo**, UOC Medicina Interna, Ospedale di Rovigo; **Mauro Pinelli, Daniela Mastroiacovo** UOSD Angiologia e Diagnostica Vascolare, Ospedale SS Filippo e Nicola, Avezzano (L’Aquila); **Walter Ageno, Giovanna Colombo**, UO. Medicina I, Ospedale di Circolo, Varese; **Eugenio Bucherini**, SS Medicina Vascolare- Angiologia, Ambulatorio Anticoagulanti, Ospedale Civile di Faenza, Faenza (Ravenna); **Domizio Serra**, Centro Trombosi, Ospedale Evangelico Internazionale, Genova; **Andrea Toma, Pietro Barbera**, UOC di Patologia Clinica, Ambulatorio Terapia Anticoagulante Orale, O.C. "L.Cazzavillan" Arzignano, (Vicenza); **Carmelo Paparo**, Patologia Clinica, Ospedale Maggiore Chieri (Torino); **Antonio Insana**, SC Patologia Clinica, Ospedale Santa Croce Moncalieri, Moncalieri (Torino); **Serena Rupoli**, Clinica Ematologica,AOU- Ospedali Riuniti Ancona, Ancona; **Giuseppe Malcangi**, Centro Emofilia e Trombosi, Policlinico di Bari, Bari; **Maddalena Loredana Zighetti**, SIMT- AO San Paolo, Milano; **Catello Mangione**, Sezione Trasfusionale Ospedale di Galatina, Galatina (Lecce); **Domenico Lione**, UOC Patologia Clinica, Ospedale Perrino, Brindisi; **Paola Casasco**, Servizio Medicina Trasfusionale Tortona (Alessandria); **Giovanni Nante**, UOS Geriatria ULSS 16 Padova, Padova; **Alberto Tosetto**, Divisione di Ematologia, Ospedale San Bortolo, Vicenza; **Vincenzo Oriana**, Centro Emofilia, Presidio Ospedaliero Morelli- Reggio Calabria; **Nicola Lucio Liberato**, UO Medicina Interna Ospedale di Casorate Primo, Pavia.
